# Role of Moesin Phosphorylation in Retinal Pericyte Migration and Detachment Induced by Advanced Glycation Endproducts

**DOI:** 10.3389/fendo.2020.603450

**Published:** 2020-11-18

**Authors:** Shuang-Shuang Zhang, Jia-Qing Hu, Xiao-Hui Liu, Li-Xian Chen, Hong Chen, Xiao-Hua Guo, Qiao-Bing Huang

**Affiliations:** ^1^ Guangdong Provincial Key Lab of Shock and Microcirculation, Department of Pathophysiology, School of Basic Medical Sciences, Southern Medical University, Guangzhou, China; ^2^ The First School of Clinical Medicine, Southern Medical University, Guangzhou, China; ^3^ Department of Endocrinology, Zhujiang Hospital, Southern Medical University, Guangzhou, China; ^4^ Trauma Care Center, Third Affiliated Hospital of Southern Medical University, Guangzhou, China

**Keywords:** advanced glycation endproducts, rat retinal microvascular pericyte, moesin, CD44, migration, immature neovascularization

## Abstract

Proliferative diabetic retinopathy (PDR) involves persistent, uncontrolled formation of premature blood vessels with reduced number of pericytes. Our previous work showed that advanced glycation endproducts (AGEs) induced angiogenesis in human umbilical vein endothelial cells, mouse retina, and aortic ring, which was associated with moesin phosphorylation. Here we investigated whether moesin phosphorylation may contribute to pericyte detachment and the development of PDR. Primary retinal microvascular pericytes (RMPs) were isolated, purified from weanling rats, and identified by cellular markers α-SMA, PDGFR-β, NG2, and desmin using immunofluorescence microscopy. Effects of AGE-BSA on proliferation and migration of RMPs were examined using CCK-8, wound healing, and transwell assays. Effects on moesin phosphorylation were examined using western blotting. The RMP response to AGE-BSA was also examined when cells expressed the non-phosphorylatable Thr558Ala mutant or phospho-mimicking Thr558Asp mutant of moesin or were treated with ROCK inhibitor Y27632. Colocalization and interaction between CD44, phospho-moesin, and F-actin were observed. Experiments with cultured primary RMPs showed that AGE-BSA inhibited the proliferation, enhanced the migration, and increased moesin phosphorylation in a dose- and time-dependent manner. AGE-BSA also triggered the rearrangement of F-actin and promoted the interaction of CD44 with phospho-moesin in RMPs. These effects were abrogated in cells expressing the non-phosphorylatable moesin mutant and the application of ROCK inhibitor Y27632 attenuated AGE-induced alteration in cultured RMPs by abolishing the phosphorylation of moesin. However, those AGE-induced pathological process occurred in RMPs expressed the phospho-mimicking moesin without AGE-BSA treatment. It is concluded that AGEs could activate ROCK to mediate moesin phosphorylation at Thr558, and resulting phospho-moesin interacts with CD44 to form CD44 cluster, which might stimulate the migration of RMPs and subsequent RMP detachment in microvessel. This pathway may provide new drug targets against immature neovessel formation in PDR.

## Introduction

Diabetic retinopathy is one of the most common complications of diabetes, affecting roughly one third of adults with diabetes and causing a large proportion of cases of adult blindness (1–3). Symptoms of diabetic retinopathy include blurred vision, the appearance of dark spots, the perception of “floaters” in the field of vision, eye pain, double vision, reduction in low-light perception, sudden vision loss, and even complete blindness (4). The non-proliferative form of diabetic retinopathy involves microaneurysm formation and intraretinal hemorrhage but not abnormal retinal neovascularization. This form can progress to proliferative diabetic retinopathy (PDR), in which proliferation of endothelial cells leads to uncontrolled neovascularization and sprouts in the retina. This can lead to blood leakage from immature vessels into the vitreous, greatly increasing the probability of vision loss (4, 5).

Pericytes help to ensheath the retinal microvasculature and protect endothelial cells from hypoxic insults and angiogenic stimuli (6). The early stage of PDR involves the loss of retinal pericytes when pericytes undergo apoptosis or migrate into the perivascular parenchyma. Pericytes are considered critical for microvascular control (7). During normal angiogenesis, two sprouts join and initiate blood flow in the newly formed loop, and subsequent interactions between endothelial cells and pericytes trigger the construction of new basement membrane, leading to vessel maturation and stabilization (8, 9). Loss of pericytes leads to capillary failure and chronic hypoxia, followed by aberrant neovascularization (4, 10–12). The resulting neovessels are malformed and show a markedly increased permeability and propensity to rupture (13), which triggers an even greater extent of aberrant angiogenesis in a vicious cycle.

Advanced glycation end products (AGEs), the biochemical end products of non-enzymatic glycosylation (14), induce pericyte loss, and up-regulate vascular endothelial growth factor (VEGF) (15), allowing endothelial cells to proliferate and thereby facilitating excessive angiogenesis. This may explain why AGEs are implicated in PDR (16–18). Studies are needed to elucidate how AGEs induce pericyte loss, which may partly involve apoptosis (19). It is also necessary to further verify if AGEs play a role in low pericyte coverage of immature neovessel during the development of PDR (20, 21). Such studies might provide some new clues for the management of PDR.

As an important members of ezrin/radixin/moesin protein family (ERM), moesin helps to regulate cell shape and migration by linking filamentous actin to membrane proteins, such as CD44, on the cell surface (22–24). ERM is of great relevance in the organization of the cytoskeleton, serving as cross linkers between the cytoskeleton and plasma membrane through binding sites for membrane molecules on the N-terminal domain (4.1 protein and ERM [FERM]) and actin-binding sites on the COOH terminus (25, 26). The COOH-terminal domain may form an intramolecular band to the NH2-terminal 4.1 ERM homology domain or may bind to F-actin, depending on the phosphorylation state of a conserved threonine residue (Thr567 in ezrin, Thr564 in radixin, and Thr558 in moesin) (27). Our previous studies have indicated the role of moesin Thr558 residue (T558) phosphorylation in AGE-induced angiogenesis and neovessel immaturation *in vivo* and *ex vivo* mouse models and the drop out of pericytes from retinal microvessel and the detachment of pericytes in neovessel have also been observed in AGE-treated mice and aortic rings (28). It is interesting to elucidate whether moesin and its phosphorylation also occur in pericytes and play a role in AGE-induced pericyte dysfunction.

It has been revealed that a specific receptor for AGEs (RAGE) is critical in AGE-induced cellular responses. RhoA kinase (ROCK) is a typical upstream activator of moesin phosphorylation. Our previous studies have also shown that AGE-induced activation of RAGE-RhoA/ROCK signaling pathway targets moesin and plays a important role in AGE-induced moesin T558 phosphorylation and subsequent angiogenesis in vascular endothelial cells (24, 29). We speculated that this ROCK-related pathway is also involved in AGE-induced moesin phosphorylation in retinal pericytes.

CD44 is the receptor molecule of extracellular matrix protein and polysaccharide, as well as an important regulator in the process of angiogenesis. CD44’s functional state of scatter or cluster in the cells might play a critical role in the formation of heterogeneous junction between endothelial cells and pericyte during the maturation of neovessels (30, 31). We hypothesized that the interaction between moesin and CD44 might be perturbed by AGE stimulation and further affects the attachment of pericyte with endothelial cell in neovessel.

## Materials and Methods

### Chemicals

Fetal bovine serum (FBS), trypsin, penicillin, streptomycin, and Dulbecco’s modified Eagle’s medium (DMEM) were from Gibco BRL (Grand Island, NY, USA). Rabbit antibodies against moesin phosphorylated on Thr558, desmin, and CD44 were from Abcam (Cambridge, UK). Mouse antibody against total moesin and the FLAG epitope were from Cell Signaling Technology (Beverly, MA, USA). Antibody targeting NG2-Cy3 conjugate was purchased from Millipore (St. Louis, MO, USA). The following antibodies were from Santa Cruz (CA, USA): rabbit anti-PDGFR-β, mouse anti-GFAP and mouse anti-von Willebrand factor (vWF).

Secondary antibodies for immunoblotting were manufactured by Sigma (St. Louis, MO, USA). FITC-anti-rabbit IgG second antibody was from Molecular Probes (Life Technologies, Carlsbad, CA, USA) and mouse anti-α-SMA was from Sigma. ROCK inhibitor Y27632 was from TargetMol (USA). The Cell Counting Kit (CCK)-8 was from Dojindo Laboratories (Kumamoto, Kyushu, Japan). Other chemicals were from Sigma unless otherwise indicated.

### Preparation of Advanced Glycation Endproduct-Bovine Serum Albumin

AGEs in all experiments were administered in the form of AGE-BSA, prepared *in vitro* as described (32) according to the protocol (33). Briefly, bovine serum albumin (BSA; 1.75 mg/ml, pH 7.4) was incubated in phosphate-buffered saline (PBS) with d-glucose (100 mmol/L) at 37°C, while control albumin was incubated without glucose. After 8 weeks of incubation, both solutions were extensively dialyzed against PBS and purified. Endotoxin content was less than 0.5 EU/ml in both solutions based on a limulus amoebocyte lysate assay (Sigma). AGE content of AGE-BSA was 72.032 U/mg protein measured by spectrofluorometry, while AGE content of bovine serum albumin was less than 0.9 U/mg protein.

### Animals

Three-week-old male weanling rats were provided by the Laboratory Animal Centre of Southern Medical University (Guangzhou, China). All experimental procedures were conducted in accordance with the ARVO Statement for the Use of Animals in Ophthalmic and Vision Research, and were approved by the Institutional Animal Care and Use Committee of Southern Medical University.

### Isolation and Identification of Retinal Microvascular Pericytes

Primary retinal microvascular pericytes (RMPs) were obtained from retinal microvessels of 3-week-old male weanling rats as described (34). Briefly, fresh rat retinas were isolated and minced into homogeneous fragments in precooled PBS buffer. The homogenates were then suspended and incubated in 0.2% type I collagenase at 37°C for 20 min. To stop digestion, DMEM containing low glucose (5 mmol/L) and 20% FBS (L-DMEM-20) was added, the suspension was mixed gently, then filtered sequentially through 100-μm and 55-μm filters. The final filtrate was collected and centrifuged at 500 g for 5 min at 4°C. The precipitated pellets were re-suspended in DMEM containing 20 mmol/L glucose and 20% FBS, then seeded in culture dishes. After 72 h of incubation, the dishes were rinsed to remove loosely adherent cellular contamination, and the medium was replaced with L-DMEM-20 on day 3–5. When cells reached 80–90% confluence, they were digested with trypsin, and digestion was halted after 1–2 min when contaminating cells began to detach. The detached cells were removed by gently swirling the dish, discarding the medium, and adding new trypsin to cells for passage. The identity and homogeneity of RMPs were assessed based on positive staining for antibodies against α-SMA, PDGFR-β, NG2, and desmin. Endothelial and glial cells were ruled out based on negativity of vWF or GFAP, respectively. RMPs can be successfully cryopreserved and recultured without loss of typical features; they can be repeatedly passaged nine times without obvious loss of characteristic phenotype.

### Retinal Microvascular Pericyte Viability Assay

Cell viability was assessed using the CCK-8 kit. RMPs in 96-well plates were treated as described, then the medium was replaced with 10% CCK-8 solution for 3 h at 37°C. The absorbance was measured at 450 nm. RMP proliferation was evaluated directly based on optical density (OD).

### Retinal Microvascular Pericyte Migration Assay

Cell migration was assessed using scratch wound healing and transwell assays. In the scratch assay, 5 × 10^5^ cells were cultured in 24-well plates for 48 h in complete medium in order to form monolayer. The monolayer was scratched using a 10-μl pipette tip to leave a linear wound, then treated as described for 24 h at 37°C. Images were captured immediately and also at 24 h after treatment as described. In three fields of view per slide, RMP migration was calculated as (open image area at 24 h/initial open image area) x 100%.

In the transwell assay, 100 μl cell suspension at 5 × 10^5^/ml was plated in the upper chamber of a transwell plate (Corning, NY, USA) containing a filter with 8-μm pores. Then 500 μl of fresh medium containing 100 μg/ml BSA or AGE-BSA (25, 50, 100 μg/ml) were added to the upper chamber medium. Culture medium was added to the lower chamber as chemoattractant, and cells were incubated for 24 h at 37°C. Migrating cells passed through the polycarbonate film, while non-migrating cells were wiped away using swabs. The migrated cells were stained with crystal violet and then photographed and counted with a microscope.

### Western Blot Analysis

Total cellular extracts were lysed with lysis buffer on ice and sonicated briefly. Protein sample concentrations were measured by BCA protein assay kit. Samples were separated by sodium dodecyl sulfate-polyacrylamide gel electrophoresis (SDS-PAGE) and transferred to polyvinylidene fluoride (PVDF) membranes. Membranes were blocked with 5% BSA in TBS containing 0.5% Tween 100 (TBS-T) for 1 h at room temperature. Incubation with relative primary antibody was performed overnight at 4°C on a rocker, followed by incubation with horseradish peroxidase (HRP)-conjugated secondary antibody for 1 h at room temperature. Protein bands were visualized by chemiluminescence. Densitometric analysis was performed using a Kodak IS2000R Imaging Station.

### Co-Immunoprecipitation of CD44 and Phospho-Moesin

RMPs were washed three times in precooled PBS and resuspended in lysis buffer on ice for 20 min. Lysates were centrifuged at 12,000 *g* at 4°C for 15 min. Then the supernatant was incubated with goat antibody against CD44 at 4°C overnight. Prewashed protein A/G beads were added to the mixture and incubated for 3 h at 4°C on a rotator. After centrifugation, beads were washed five times with lysis buffer. Isolated protein complexes were denatured for 5 min at 95°C, subjected to SDS-PAGE and transferred to western blotting, followed by immunoblotting with anti-phospho-moesin or anti-CD44 antibody. The bands were detected with HRP-based chemiluminescence.

### Site-Specific Mutagenesis of Moesin and Transfection of Plasmids Into Retinal Microvascular Pericytes

Based on our previous analysis of moesin mutants (24, 32), we engineered plasmids encoding a non-phosphorylatable Thr558Ala mutant of moesin (pcDNA3/FLAG-moesinThr558Ala, T558A) and a phospho-mimicking Thr558Asp mutant (pcDNA3/FLAG-moesinThr558Asp, T558D). The mutations were confirmed by nucleotide sequencing. Plasmids were purified for transfection using an EndoFree Plasmid Midiprep Kit (Omega Bio-tek, Norcross, GA, USA). RMPs were plated at 2×10^5^ cells per well in a six-well plate on the night before transfection. DNA (2 μg) was incubated with 8 μl lipofectamine LTX and 2 μl Plus Reagent (Invitrogen, Carlsbad, CA, USA) in 500 μl Opti-MEM at room temperature for 30 min. The cultured cells were washed once with Opti-MEM, incubated with the DNA-lipid complexes for 48 h, then stimulated with AGE-BSA (100 μg/L, 24 h).

### Immunofluorescence Microscopy

Gelatin-coated glass-bottomed microwell plates (MatTek, MA, USA) were used to culture RMPs as described above. Cells were fixed for 10 min at room temperature in PBS containing 4% (w/v) paraformaldehyde, then permeabilized in 0.5% (w/v) Triton X-100. The cell layers were washed in PBS twice and blocked in 5% BSA for 1 h. After overnight incubation with 100 μl of primary antibody against phospho-moesin (diluted 1:200) or CD44 (diluted 1:100), the cells were washed three times with PBS before incubation with 100 μl of a 1:200 dilution of FITC- or rhodamine-conjugated secondary antibody in PBS containing 5% (w/v) BSA for 1 h at room temperature. In the case of F-actin staining, rhodamine-phalloidin was used at a concentration of 2 U/ml in PBS. After 2 h of incubation, the specimens were again washed three times with PBS. Cells were further incubated with diamidino-2-phenylindole (DAPI, 1:1,000) for 15 min and then washed with PBS. The staining results were imaged using a Zeiss LSM780 laser confocal scanning microscope (Zeiss, Germany).

### Statistical Analysis

Data were normalized to control values and expressed as mean ± SD. The results were analyzed by one-way analysis of variance (ANOVA) followed by Tukey’s test. The level of significance was set at *P* < 0.05.

## Results

### Identification of Retinal Microvascular Pericytes in Primary Cultures

**Figure 1:**
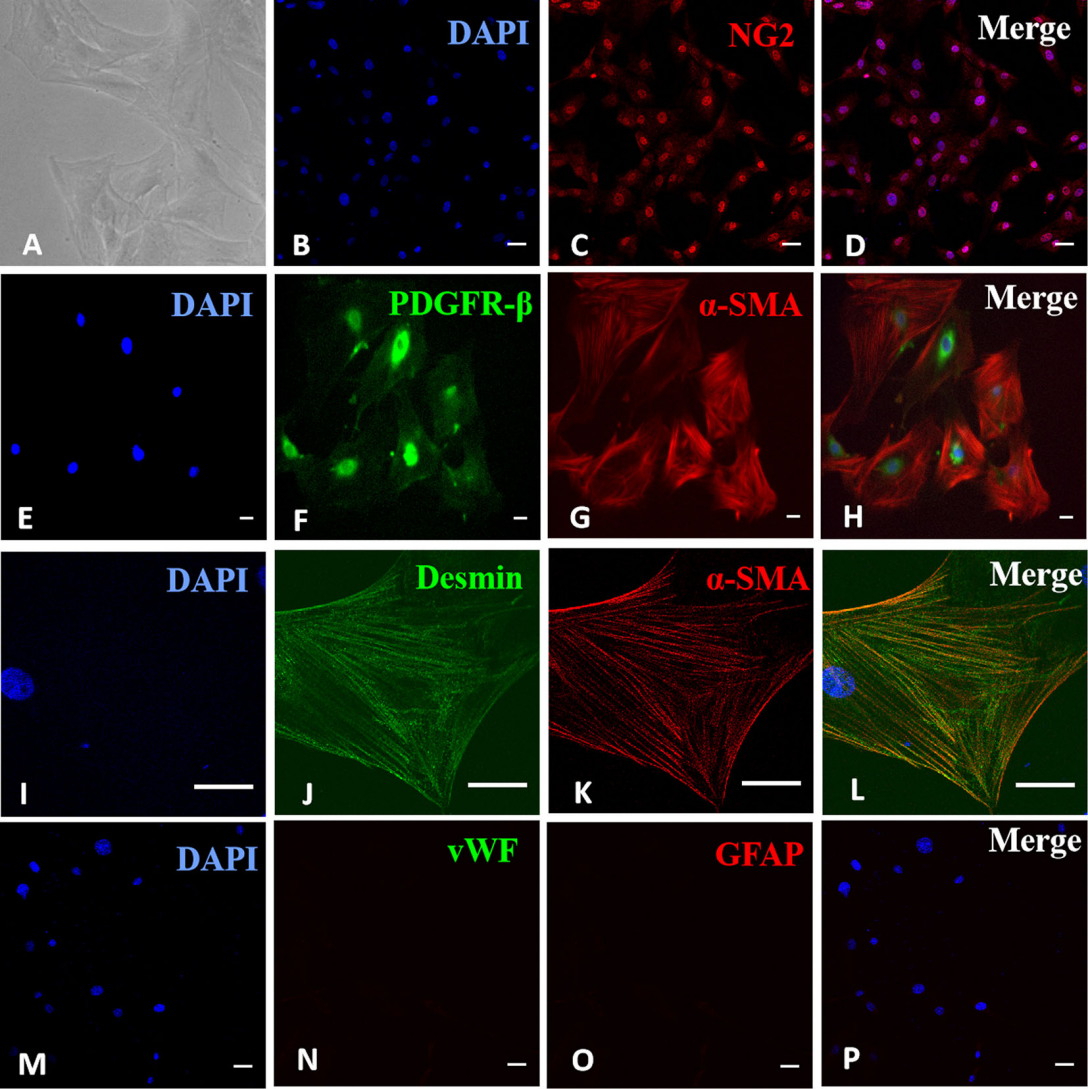
Identification of retinal microvascular pericytes (RMPs) in primary culture. **(A)** Sparsely spreading cells had large, flat, irregularly triangular bodies with several long processes. **(B–D)** Positive immunostaining for NG2. **(E–H)** Positive double immunostaining for α-SMA and PDGFR-β. **(I–L)** Positive double immunostaining for α-SMA and desmin. **(M–P)** Negative staining for vWF and GFAP. Scale bar, 30 μm. **Figure S1** revealed the purity of RMPs in culture with positive staining for desmin in most cells.

Cells were identified as RMPs under an optical microscope based on typical pericyte morphology of irregular shape, long processes, and large, flat cell bodies (**Figure 1A**). Their identities were confirmed based on positive immunostaining of pericyte markers NG2 (**Figures 1B-D**), α-SMA, and PDGFR-β (**Figures 1E–H**) as well as desmin **(** **Figures 1I–L**). The possibilities of contaminating endothelial cells and glial cells were ruled out by negative staining of endothelial cell marker vWF or glial marker GFAP (**Figures 1M–P**). Most cells stained positive for desmin (listed in **Figure S1**), confirming the purity of RMPs in culture.

### Dose- and Time-Dependent Effects of Advanced Glycation Endproduct-Bovine Serum Albumin on Retinal Microvascular Pericyte Viability and Migration

**Figure 2:**
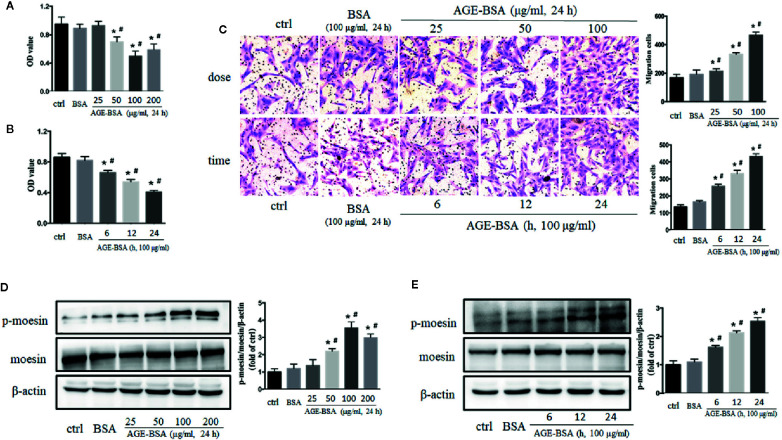
The dose- and time-dependent effects of advanced glycation-bovine serum albumin (AGE-BSA) treatment on retinal microvascular pericyte (RMP) viability, moesin phosphorylation. **(A, B)** The effects of AGE-BSA treatment on RMP viability. **(C)** The effects of AGE-BSA treatment on RMP migration. **(D, E)** The effects of AGE-BSA treatment on moesin phosphorylation in RMPs. Data shown are representative of experimental and quantitative results. N = 3 independent experiments. *p < 0.05 *vs*. control, ^#^ *p* < 0.05 *vs*. BSA. **Figure S2** confirmed the expression of moesin and ERM proteins in RMPs by positive staining of immunofluorent moesin or ERM protein in most cells.

Cultured RMPs were incubated with gradient concentrations of AGE-BSA (25, 50, 100, and 200 μg/ml) for 24 h, and then assayed for proliferation ability using CCK-8 kit. AGE-BSA reduced RMP viability in a dose-dependent manner relative to the viability of untreated and BSA-treated cells (**Figure 2A**). Viability was then measured in cultured RMPs treated with 100 μg/ml AGE-BSA for 6, 12, or 24 h, respectively. The results showed that RMP viability was decreased in a time-dependent manner (**Figure 2B**). On the contrary, AGE-BSA significantly increased the migration of RMPs in a dose- and time-dependent fashion (**Figure 2C**).

### The Application of Advanced Glycation Endproduct-Bovine Serum Albumin Induces Moesin Phosphorylation in Retinal Microvascular Pericytes

The expression of moesin in RMPs was clarified by positive staining of immunofluorent moesin or ERM protein (listed in **Figure S2**) and western blotting (**Figures 2D, E**). The application of AGE-BSA in gradient concentration and different timing induced dose- and time-dependent phosphorylation of moesin in RMPs (**Figures 2D, E**). Based on these studies, 100 μg/ml AGE-BSA was chosen to treat RMPs for 24 h in all subsequent experiments on moesin phosphorylation.

### Advanced Glycation Endproduct-Induced Migration of Retinal Microvascular Pericytes Involves Moesin Phosphorylation at Thr558

**Figure 3:**
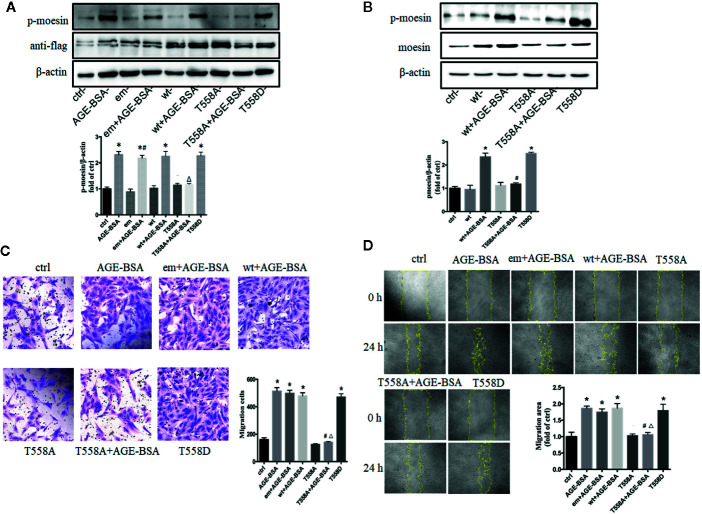
Advanced glycation-bovine serum albumin (AGE-BSA)-induced migration of retinal microvascular pericytes (RMPs) involves moesin phosphorylation at Thr558. RMPs were transfected with empty vector (em), wild type (wt) moesin plasmid, pcDNA3/FLAG-moesinThr558Ala (T558A), respectively, for 24 h with or without AGE-BSA (100 μg/ml), treatment. pcDNA3/FLAG-moesinThr558Asp (T558D) was transfected into RMPs without AGE-BSA treatment. **(A)** The moesin phosphorylation and expression of anti-flag in RMPs were detected using western blotting. *p < 0.05 *vs*. control, ^#^ *p < *0.05 *vs*. empty vector, ^△^ *p < *0.05 *vs*. AGE-BSA. **(B)** Total moesin expression and moesin phosphorylation in RMPs were detected using western blotting. *p < 0.05 *vs*. control, ^#^ *p* < 0.05 *vs*. wt+AGE-BSA. The cropped images represent blotting experiments that were performed under the same experimental conditions. **(C, D)** Cell migration was detected using scratch wound healing and transwell assays. N = 3 independent experiments. *p < 0.05 *vs*. control, ^#^ *p* < 0.05 *vs*. AGE-BSA, ^△^ *p* < 0.05 *vs*. wt+AGE-BSA.

Our previous studied has indicated that moesin phosphorylation on Thr558 is required for AGE-induced human umbilical vein endothelial cell migration and tube formation (24). To examine whether AGE-induced pericyte migration involves the same phosphorylation event, we transfected RMPs with expression plasmids encoding a Thr558Ala mutant of moesin that cannot be phosphorylated, or a Thr558Asp mutant that mimics the phosphorylated state of moesin, and then we examined how AGE-BSA affected RMP migration in each case. To confirm that this experimental system was working, we first verified the successful transfection of plasmids with anti-flag band (**Figure 3A**) and then detected the expression and phosphorylation of moesin after plasmid transfection (35). The results showed that, although the basic expression of moesin was increased by wt plasmid transfection, the level of p-moesin was not changed, while only the application of AGE-BSA enhanced the level of p-moesin along with the over expression of moesin (**Figures 3A, B** **)**. We further verified that cells overexpressing Thr558Ala mutant showed lower moesin phosphorylation at Thr558 following AGE-BSA treatment, while cells overexpressing Thr558Asp showed higher levels of moesin phosphorylation at Thr558 (**Figures 3A, B** **)**. We further examined the effect of each mutation on RMP migration. The Thr558Asp mutation alone led to similar migration area in wound healing assay and similar proportion of migrated cells in transwell migration assay as AGE-BSA treatment (**Figures 3C, D**). In contrast, the Thr558Ala mutation attenuated AGE-induced RMP migration. These results indicate that the Thr558 is the phosphorylation site in AGE-induced moesin activation and Thr558 phosphorylation results in subsequent RMP migration.

### Rho-Associated Protein Kinase Is Involved in Advanced Glycation Endproduct-Induced Moesin Phosphorylation and Retinal Microvascular Pericyte Migration

**Figure 4:**
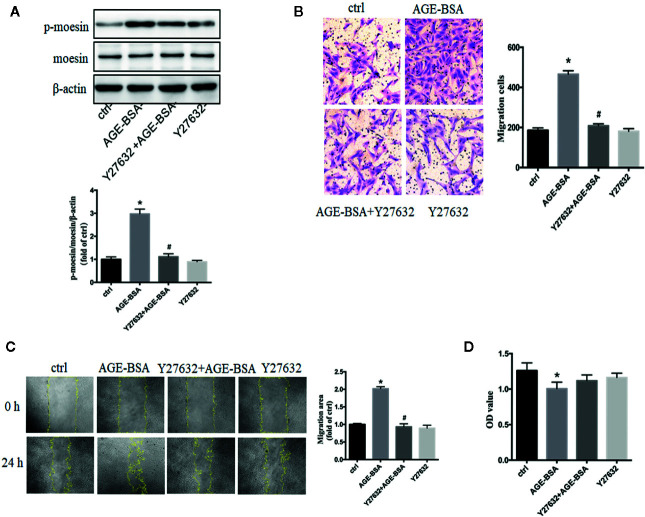
Advanced glycation-bovine serum albumin (AGE-BSA)-induced moesin phosphorylation and RMP migration involve Rho-associated protein kinase (ROCK). Y27632, specific inhibitor of ROCK (10 μmol/L), was pre-incubated 1 h before AGE-BSA (100 μg/ml, 24 h) application. **(A)** Moesin phosphorylation and total moesin expression in RMPs were detected using immunoblotting. The cropped images represent blotting experiments that were performed under the same experimental conditions. **(B, C)** Cell migration was detected using scratch wound healing and transwell assays. **(D)** Cell viability. Results shown are representative experiment and quantitative results. N = 3 independent experiments. *p < 0.05 *vs*. control, ^#^ *p < *0.05 *vs*. AGE-BSA.

In human umbilical vein endothelial cells, the activation of RhoA/ROCK pathway participated in the process of AGE-induced moesin phosphorylation, endothelial hyperpermeability, and angiogenesis (24, 29, 32, 36, 37). In present study, specific ROCK inhibitor Y27632 was used to inhibit RhoA/ROCK activity in RMPs. ROCK inhibition significantly attenuated AGE-induced moesin phosphorylation (**Figure 4A**) and migration (**Figures 4B, C**), without affecting cell viability in the presence or absence of AGE-BSA (**Figure 4D**). These results suggest that activation of RhoA/ROCK might play a critical role in AGE-induced moesin phosphorylation and RMP migration.

### Moesin Phosphorylation Triggers Formation of Actin Stress Fibers That Colocalize With the Phospho-Moesin

**Figure 5:**
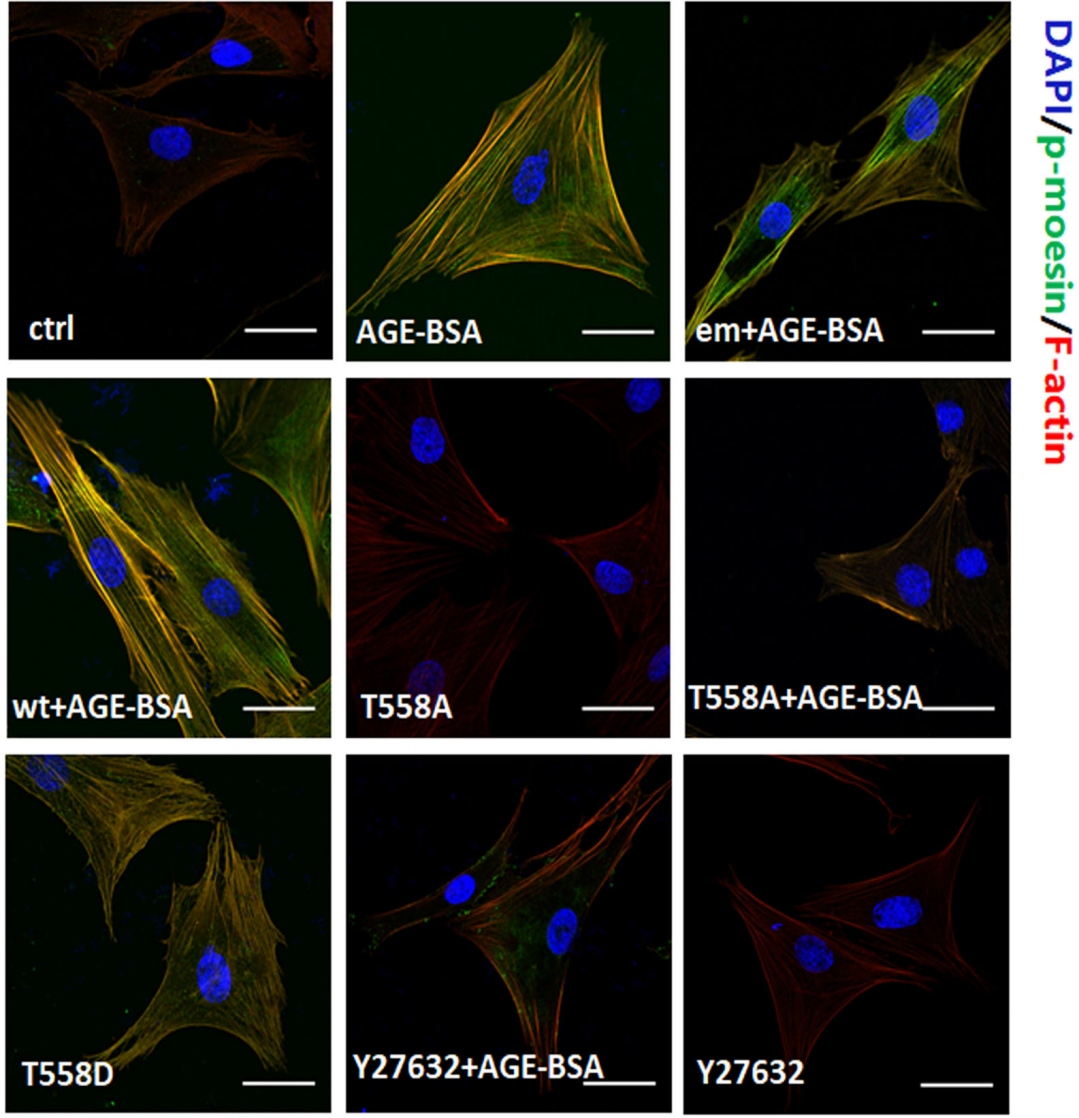
Moesin phosphorylation triggers formation of actin stress fibers that colocalizes with the phospho-moesin. Phospho-moesin was identified by immunostaining with a primary antibody, followed by FITC-conjugated secondary antibody (green). Rhodamine-phalloidin and DAPI were used to stain F-actin (red) and nuclear DNA (blue), respectively. Results shown are representative images in at least three experiments. Scale bar, 30 μm.

In untreated RMPs, F-actin localized mainly around the cellular cortex and the staining of phospho-moesin was weak (**Figure 5**). Stimulation with AGE-BSA led to reorganization of cortical filaments, giving rise to elongated stress fibers and strong staining of phospho-moesin that colocalized with the newly formed F-actin. Similar results were obtained either by expressing Thr558Asp mutant of moesin or by expressing the wild-type or endogenous moesin and then stimulating with AGE-BSA. Conversely, expression of Thr558Ala mutant of moesin prevented these AGE-induced reorganization of F-actin, and similar results were observed when RMPs were treated with ROCK inhibitor Y27632. These results suggest that activation of RhoA/ROCK pathway and moesin phosphorylation at Thr558 are involved in AGE-induced pericyte mobility and subsequent migration.

### Advanced Glycation Endproducts Up-Regulate Expression of CD44 and Its Binding to Phospho-Moesin

**Figure 6:**
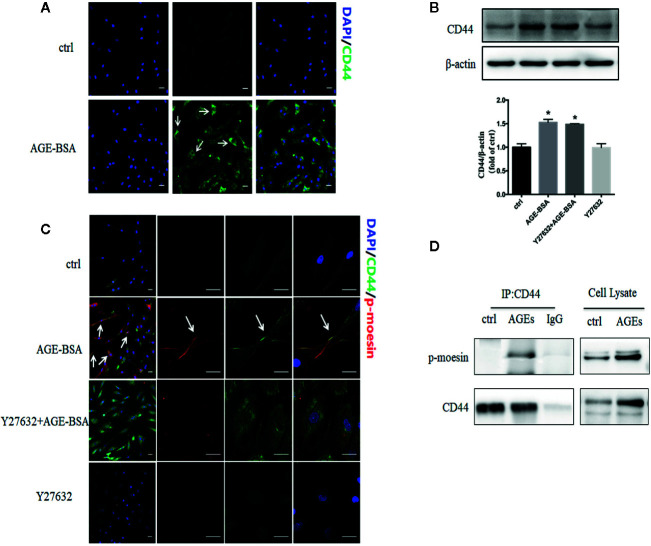
Advanced glycation-bovine serum albumin (AGE-BSA) up-regulates the expression of CD44 and its binding to phospho-moesin. **(A)** Cultured retinal microvascular pericytes (RMPs) were treated with AGE-BSA (100 μg/ml, 24 h) and subjected to immunostaining with antibodies to CD44 (green). **(B, C)** Cultured RMPs were treated with AGE-BSA or Y27632 + AGE-BSA and the expression of CD44 was detected using immunoblotting. CLSM colocalization studies were performed for phospho-moesin (red) and CD44 (green). **(D)** RMPs treated with or without AGE-BSA were lysed and subjected to immunoprecipitation (IP) with antibodies against CD44 or with control IgG. The resulting precipitates as well as the cell lysates were subjected to immunoblot analysis with antibodies against phospho-moesin or CD44. Results shown are representative experiment and quantitative results. N = 3 independent experiments. *p < 0.05 *vs*. control. Scale bar, 30 μm.

Activated ERM proteins bind to CD44, facilitating their cross-linking with actin filaments and the formation of heterogeneous junctions with other cell types (26). In our unstimulated RMP cultures, CD44 showed weak, diffuse distribution throughout the cytoplasm (**Figure 6A**). Stimulation with AGE-BSA increased CD44 expression and led to the formation of intense dots at the cell edge. Western blots confirmed that AGE-BSA increased CD44 expression, and this up-regulation did not require ROCK activity (**Figure 6B**). The CD44 at the cell edge colocalized with phospho-moesin (**Figure 6C**), and the interaction of these two proteins was confirmed by showing that CD44 was precipitated by an antibody against phospho-moesin but not by an IgG control (**Figure 6D**). These results suggest that AGEs trigger binding of phospho-moesin to CD44 in RMPs.

## Discussion

We have previously demonstrated (28), *in vivo* and *ex vivo*, that AGEs promoted immature neovascularization in the mouse retina and aortic ring, as well as induced RMP loss and detachment from microvessels. Based on it, we have shown here in further, that this RMP detachment involves phosphorylation of moesin at Thr558. AGE initiates a series of steps that lead to the interaction of phospho-moesin not only with F-actin but also CD44, the formation of stress fiber and the migration of RMP. This series of steps requires the activation of ROCK.

Various stress inducers, such as ischemia, hypoxia, injury, and AGE exposure, cause pericytes to detach and migrate from vessels into the perivascular parenchyma (38, 39). In a rat model of middle cerebral artery occlusion, pericytes were found to detach from basal lamina within 1 h after stroke, then migrate toward the hypoperfusion lesion (40). During traumatic spinal cord injury in mice, pericytes detach from the basal lamina of the cerebrovasculature and migrate through the extracellular matrix to the area surrounding the site of injury (41). In ischemic retinopathy, high PDGF-β level in RMPs leads to NCK1/2-dependent pericyte migration, which promotes abnormal angiogenesis and inhibits retinal revascularization. Inhibition of PDGF-β or downstream NCK1/2 signaling blocks pericyte migration and pathological neovascular tufts, stabilizing retinal vessels (42). AGE promotes not only pericyte migration but also their loss through apoptosis (43–46). Both processes are implicated in diabetic retinopathy. Pericyte loss is already detectable after 3 months of diabetes in experimental models (47), while pericyte apoptosis usually becomes detectable at later stages (48).

The two processes of apoptosis and migration may involve different subpopulations of RMPs, which have diverse origins in the neural crest, hematopoietic cells, and endothelial cells (49). The presence of different RMP subpopulations may explain, for example, why hyperglycemia-induced pericyte migration in a mouse model of diabetes is restricted to straight capillaries of the retinal microvasculature (21). The diversity of RMPs is the likely reason why no pan-pericyte marker has been identified. We identified RMPs using a panel of markers (α-SMA, desmin, NG2, PDGFR-β), since none of the markers on its own is sufficient to recognize all pericytes (6, 50–52). For instance, NG2 proteoglycan can be expressed in macrophages (53) and is not expressed by all pericytes (54), while PDGFR-β is a known marker of fibroblasts (55). The diversity of RMPs implies that they respond differently to chronic hyperglycemia in different patients. Further experiments are needed to address what proportions of pericyte loss are due to migration or apoptosis, and to track the fate of pericytes that migrate away.

Our finding that AGEs triggers pericyte detachment and migration from microvasculature is consistent with previous studies (20, 21) examining AGE-induced pericyte migration, while the mechanism is not fully understood. The Ang-2/Tie-2 signaling pathway has been illustrated on RMP migration in the diabetic retina in XLacZ mice (21), and the absence of Ang-2 restores vessel integrity and recruits pericytes to vessels (56, 57). In another study, AGE-BSA appears to promote the migration of bovine RMPs *via* the RAGE-Src-ERK1/2-FAK-1-paxillin signaling pathway (20). It has been revealed that the intracellular molecular complex FERM (protein 4.1, ezrin/radixin/moesin) participates in the retina lamination, and particularly, in the formation of tight junction of retinal pigmented epithelium in zebrafish, demonstrating the involvement of ERM protein in retina structure and function (58). Our results indicate that the T558 phosphorylation of moesin and the subsequent clustering of membrane protein CD44 are involved in AGE-induced RMP migration, resulting in the detachment of pericyte from microvessel and the damage of vessel integrity.

The signaling pathways for AGE-induced cellular responses have been explored in our serious studies. Using RAGE antibody (29), dominant mutant RAGE (59), and RAGE knockout mice (28, 60), respectively, we have demonstrated that AGEs exert the effects on inducing microvascular hyperpermeability and immature angiogenesis by binding with RAGE, which results in subsequent activation of RhoA-ROCK pathway. Activated ROCK could interact with moesin and enhance AGE-induced moesinT558 phosphorylation (32). While the express of RAGE has been confirmed in RMPs (61), this AGE/RAGE binding is postulated in pericytes too. It have been reported that ROCK regulates moesin function (62) and actin cytoskeleton organization (63). Inhibition of ROCK signaling inhibits actin remodeling and ERM phosphorylation in human colonic epithelial cells (64). The results in present study indicate that AGEs induce RMP migration by triggering the interaction between phospho-moesin and CD44 in a process that also requires ROCK activity for moesin Thr558 phosphorylation. Taken together, these findings suggest that AGE-induced RMP migration is related to decreasing cell-cell contacts and increasing cell motility, which requires cytoskeletal reorganization.

Our results implicating the interaction between phospho-moesin and CD44 in pericyte loss extend the list of processes in which interactions between phosphorylated ERM proteins and CD44 drive changes in cell-cell contacts. These interactions, for example, lead to loss of cell-cell contacts in epithelial-mesenchymal transition of retinal pigment epithelial cells (65) and during the generation of myofibroblasts (66). Glioma progression involves the interaction of phospho-moesin with CD44 and the Wnt-β-catenin pathway (67). Phospho-ERM binds to CD44 in a single pseudopod in myeloid cells (68), and the CD44 binds in turn to hyaluronan in the extracellular matrix (69, 70). Interaction between hyaluronan and CD44 in cancer decreases endothelial cell-cell contacts (71), leading to endothelial cells barrier disruption, which is an initial event of aberrant angiogenesis in tumor. Future studies should co-culture pericytes and endothelial cells in order to examine the role of phospho-moesin/CD44 complexes in the recruitment of pericytes to endothelial cells. Another interesting result is the enhancement of CD44 expression by AGE-BSA treatment, which deserves to be further explored.

**Figure 7:**
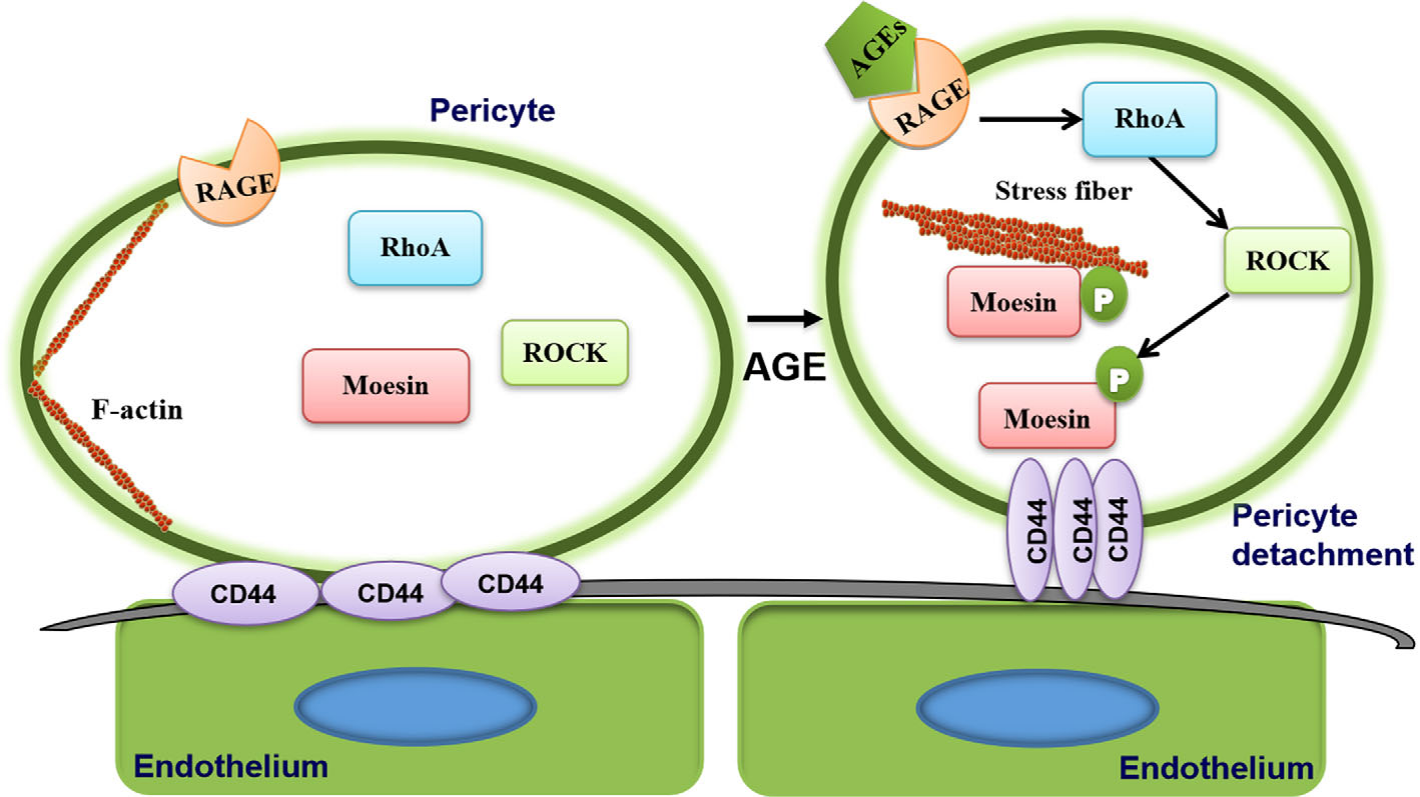
Proposed mechanism of advanced glycation endproduct (AGE)-induced pericyte detachment. AGE triggers moesin phosphorylation at Thr558 *via* a ROCK-mediated pathway, which promotes interaction between the phospho-moesin and CD44, leading to reorganization of the actin cytoskeleton, ultimately resulting in AGE-induced pericyte drop out. The resulting reduction in contact between endothelium and pericytes and reduced recruitment of pericytes may contribute to aberrant angiogenesis in proliferative diabetic retinopathy (PDR).

The present study was motivated in large part by our previous observation that AGE-induced moesin phosphorylation promotes proliferation, migration, and tube formation by human umbilical vein endothelial cells, leading to excessive angiogenesis (24). Recently, we also demonstrated AGE-induced moesin phosphorylation induces immature angiogenesis *in vivo* and *ex vivo* mouse models (28). Our present results suggest a new mechanism of AGE-induced pericyte migration through moesin phosphorylation (**Figure 7**), resulting in less pericyte coverage and disruption of vessel integrity. AGE triggers moesin phosphorylation at Thr558 *via* a ROCK-mediated pathway, which promotes interaction between the phospho-moesin and CD44, leading to reorganization of the actin cytoskeleton. The resulting reduction in contact between endothelia and pericytes and reduced recruitment of pericytes may contribute to aberrant angiogenesis in PDR. These insights might establish a new target for the management of immature vessel formation during the development of diabetic retinopathy.

## Data Availability Statement

The datasets generated from this study will be made available by the authors.

## Ethics Statement

The animal study was reviewed and approved by Institutional Animal Care and Use Committee of Southern Medical University.

## Author Contributions

S-SZ and Q-BH conceived and designed research. S-SZ, J-QH, X-HL, and L-XC performed experiments. S-SZ analyzed data, prepared figures and drafted manuscripts. S-SZ and Q-BH interpreted results of experiments. HC, X-HG, and Q-BH edited and revised manuscript. All authors contributed to the article and approved the submitted version.

## Funding

This work was supported by National Natural Science Foundation of China Grants 81370226, 81870210, and Guangdong Basic and Applied Basic Research Foundation, 2019A1515012022.

## Conflict of Interest

The authors declare that the research was conducted in the absence of any commercial or financial relationships that could be construed as a potential conflict of interest.

## Supplementary Material

Supplementary Figure 1 Positive Staining of Desmin. Scale bar, 100 μm.

Supplementary Figure 2 Positive Staining of ERM and moesin. Scale bar, 100 μm.
