# Supplementary figures and images for: Role of Moesin Phosphorylation in Retinal Pericyte Migration and Detachment Induced by Advanced Glycation Endproducts
Source: Front Endocrinol (Lausanne). 2020 Nov 18;11:603450. doi: 10.3389/fendo.2020.603450 (PMC7708375; doi:10.3389/fendo.2020.603450)

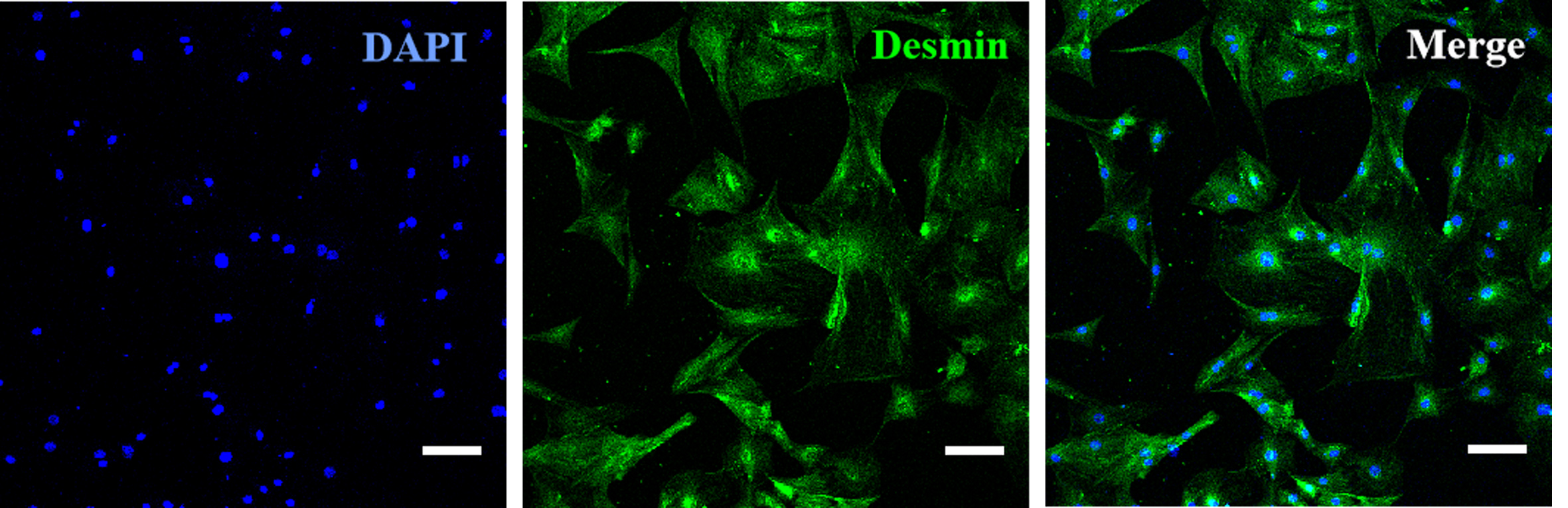

Supplement: Supplementary Figure 1 — Positive Staining of Desmin. Scale bar, 100 μm. [file Image_1.jpeg]

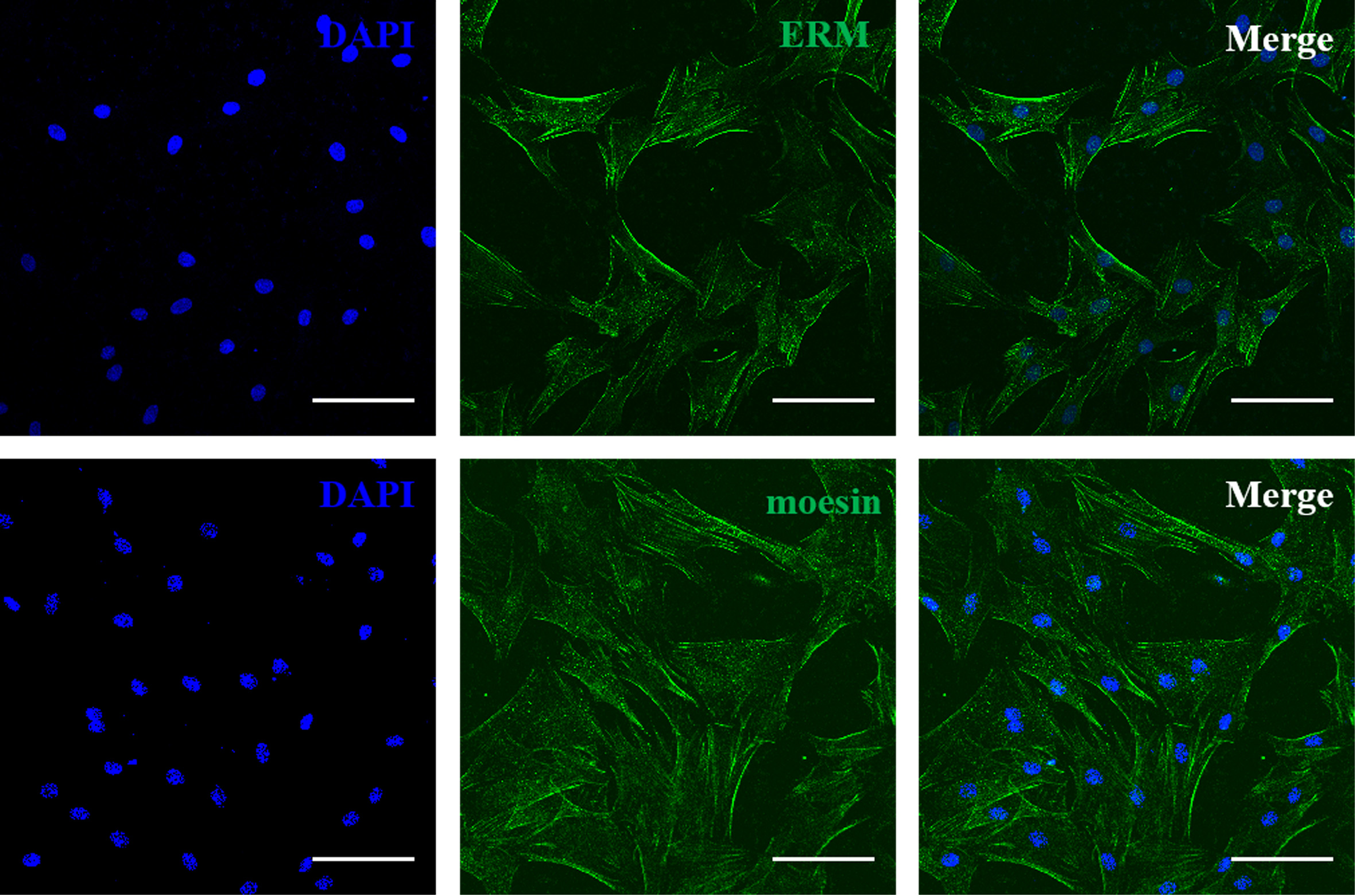

Supplement: Supplementary Figure 2 — Positive Staining of ERM and moesin. Scale bar, 100 μm. [file Image_2.jpeg]
